# Isavuconazole Exposure in Critically Ill Patients Treated with Extracorporeal Membrane Oxygenation: Two Case Reports and a Narrative Literature Review

**DOI:** 10.3390/antibiotics12071085

**Published:** 2023-06-21

**Authors:** Beatrijs Mertens, Omar Elkayal, Erwin Dreesen, Joost Wauters, Philippe Meersseman, Yves Debaveye, Karlien Degezelle, Pieter Vermeersch, Matthias Gijsen, Isabel Spriet

**Affiliations:** 1 Department of Pharmaceutical and Pharmacological Sciences, KU Leuven and Pharmacy Department, University Hospitals Leuven, 3000 Leuven, Belgium; 2 Department of Pharmaceutical and Pharmacological Sciences, KU Leuven, 3000 Leuven, Belgium; 3 Department of Microbiology, Immunology and Transplantation, KU Leuven and Medical Intensive Care Unit, University Hospitals Leuven, 3000 Leuven, Belgium; 4 Department of Cellular and Molecular Medicine, KU Leuven and Intensive Care Unit, University Hospitals Leuven, 3000 Leuven, Belgium; 5 Department of Perfusion Technology, University Hospitals Leuven, 3000 Leuven, Belgium; 6 Clinical Department of Laboratory Medicine, University Hospitals Leuven, 3000 Leuven, Belgium

**Keywords:** extracorporeal membrane oxygenation, invasive fungal infections, critical care, isavuconazole, pharmacokinetics, therapeutic drug monitoring

## Abstract

Effective dosing of isavuconazole in patients supported by extracorporeal membrane oxygenation (ECMO) is important due to the role of isavuconazole as a first-line treatment in patients with influenza- and COVID-19-associated pulmonary aspergillosis. To date, robust pharmacokinetic data in patients supported by ECMO are limited. Therefore, it is unknown whether ECMO independently impacts isavuconazole exposure. We measured isavuconazole plasma concentrations in two patients supported by ECMO and estimated individual pharmacokinetic parameters using non-compartmental analysis and two previously published population pharmacokinetic models. Furthermore, a narrative literature review on isavuconazole exposure in adult patients receiving ECMO was performed. The 24 h areas under the concentration–time curve and trough concentrations of isavuconazole were lower in both patients compared with exposure values published before. In the literature, highly variable isavuconazole concentrations have been documented in patients with ECMO support. The independent effect of ECMO versus critical illness itself on isavuconazole exposure cannot be deduced from our and previously published (case) reports. Pending additional data, therapeutic drug monitoring is recommended in critically ill patients, regardless of ECMO support.

## 1. Introduction

The mold-active triazoles voriconazole and isavuconazole are recommended as first-line therapies for invasive aspergillosis, including influenza-associated and coronavirus disease 2019 (COVID-19)-associated pulmonary aspergillosis (IAPA and CAPA, respectively) [1,2,3,4]. Antifungal treatment with voriconazole poses challenges in clinical practice due to its nonlinear pharmacokinetic (PK) profile, the risk for concentration-dependent toxicity (i.e., neuro- and hepatotoxicity) and its involvement in drug–drug interactions as a substrate and inhibitor of CYP2C19, CYP2C9 and CYP3A4 [5,6]. The newest triazole, isavuconazole, is characterized by high plasma protein binding (98–99%), high lipophilicity and a long elimination half-life (110–115 h) [7]. It undergoes hepatic metabolization via CYP3A4 and CYP3A5 iso-enzymes and—subsequently—via uridine diphosphate glucuronosyltransferases [5,7]. Compared with voriconazole, isavuconazole is a more favorable triazole in terms of PK (i.e., linear PK and thus dose-proportional exposure), safety and interaction profiles. Additionally, it has a broader antifungal spectrum, including *Mucorales* species [5,7,8]. As a consequence, isavuconazole is increasingly being used in the treatment of invasive mold infections.

The role of therapeutic drug monitoring (TDM) of isavuconazole is still controversial [2]. In the SECURE phase III clinical trial, limited interindividual variability in isavuconazole exposure was documented, and no significant exposure–response relationship for efficacy and safety was observed, suggesting that routine TDM might not be warranted [2,9,10,11,12,13]. However, phase IV studies including more diverse patient populations have revealed a higher degree of variability in exposure to isavuconazole [14,15,16,17]. This variability suggests a potential role of TDM in those patients at risk for reduced or increased exposure. TDM might be of particular importance for critically ill patients who often exhibit altered PK owing to pathophysiological changes (e.g., hypoalbuminemia and fluid shifts) and therapeutic interventions (e.g., renal replacement therapy (RRT) and extracorporeal membrane oxygenation (ECMO)) [9,18]. In clinical practice, an isavuconazole trough concentration (C_min_) between 2 mg/L and 4 mg/L is often targeted in accordance with mean isavuconazole exposure documented in the SECURE trial (i.e., mean ± standard deviation (SD) of C_min_ on days 7 and 14: 2.6 ± 1.0 mg/L and 3.0 ± 1.4 mg/L, respectively) [10,13]. However, a C_min_ of 1 mg/L, which corresponds to the European Committee on Antimicrobial Susceptibility Testing (EUCAST) breakpoint for *Aspergillus fumigatus*, *A. flavus* and *A. terreus*, might be advocated as the minimal efficacy target [19]. Although no clear exposure–toxicity relationship has been demonstrated for isavuconazole, Furfaro et al. identified a C_min_ threshold of 5.13 mg/L for toxicity, predominantly gastro-intestinal toxicity [20].

In the past years, questions have been raised regarding the impact of ECMO support on the PK of mold-active triazoles given their role in the treatment of invasive pulmonary aspergillosis in patients with virus-associated acute respiratory distress syndrome (ARDS) [3,4]. In ECMO, blood is extracted from the venous system and pumped via the extracorporeal tubing through an oxygenator, which removes carbon dioxide and oxygenates the blood before it is returned to the venous or arterial system. Thereby, ECMO allows the heart and/or lungs to be bypassed. Veno-venous ECMO provides only respiratory support, whereas veno-arterial ECMO provides both respiratory and hemodynamic support [21]. Veno-venous ECMO is increasingly used for temporary respiratory support in patients with (virus-associated) ARDS refractory to optimal medical management [22,23]. ECMO support can further complicate the challenge of effective antifungal dosing in critically ill patients as it might induce PK alterations and—accordingly—changes in drug exposure [24,25,26,27]. Increased volume of distribution, resulting from priming of the ECMO circuit, and drug sequestration into the ECMO circuit are the major factors contributing to alterations in drug exposure during ECMO support [24,25,26,27]. Based on its physicochemical characteristics (i.e., high lipophilicity and plasma protein binding), isavuconazole can theoretically be sequestered into the ECMO circuit, potentially leading to reduced plasma concentrations [5,24,25,26,27]. Currently, data on the exposure to isavuconazole in patients supported by ECMO are scarce and predominantly limited to case reports or case series. Awaiting robust data on the impact of ECMO on the PK of isavuconazole, we report isavuconazole plasma concentrations and PK parameters in two critically ill adult patients treated with isavuconazole during ECMO support. Subsequently, we compare their PK parameters and exposure metrics with previously reported ones, based on a narrative literature review.

## 2. Materials and Methods

### 2.1. Patients

Two adult patients, admitted to the intensive care unit (ICU) of the University Hospitals Leuven and concomitantly treated with isavuconazole and ECMO, were included in this case report. Isavuconazole was administered intravenously as a loading dose of 200 mg q8h during the first 48 h, followed by a maintenance dose of 200 mg q24h, according to the Summary of Product Characteristics [7]. TDM of isavuconazole was performed at the discretion of the treating physician in accordance with the hospital’s guidelines, targeting trough concentrations (C_min_) between 2 mg/L and 5 mg/L [10,12,13,20].

### 2.2. Sample Collection and Bioanalysis

In addition to routine TDM of isavuconazole, conducted per standard of care according to the hospital’s guidelines, extensive PK sampling was performed for both patients during concomitant isavuconazole and ECMO treatment. During one dosing interval between days 3 and 7 of isavuconazole treatment, arterial blood samples were collected in lithium heparin tubes immediately prior to the start of infusion (over 1 h) and at 0.5 h, 1 h, 2 h, 4 h, 6 h, 8 h and 12 h after the end of infusion. Blood samples were centrifuged within 72 h after collection (Mega Star 600 R, VWR, Leuven, Belgium; 1900 × *g*, 5 min) and plasma samples were stored at −80 °C until analysis. Total concentrations were measured for each sample using a validated liquid chromatography and tandem mass spectrometry method (Waters ACQUITY TQD tandem quadrupole UPLC/MS/MS system, Waters Corporation, Milford, MA, USA; ClinMass LC-MS/MS Complete kit Antimycotics, RECIPE Chemicals, Munich, Germany). Linearity of the calibration curve was found in the concentration range of 0.5 mg/L to 10.7 mg/L. The lower limit of quantification was 0.5 mg/L. Analytical recovery of isavuconazole and the internal standard was 102 ± 5% (mean ± SD). Coefficients of variation for intra- and interday precision and accuracy were all <15%.

### 2.3. Pharmacokinetic Analysis

For each patient, PK parameters of isavuconazole were estimated with three different PK methods using total isavuconazole concentrations (*n* = 8 per patient), measured via extensive PK sampling. Firstly, PK parameters were calculated using non-compartmental analysis (PKanalix version 2021R1, Lixoft SAS, Simulation Plus Inc., Lancaster, CA, USA). The 24 h areas under the concentration–time curve (AUC_0–24_) for a maintenance dose of 300 mg q24h (patient 1) or 200 mg q24h (patient 2) were estimated based on the log-linear trapezoidal rule. Clearance (CL) was calculated as dose/AUC_0–24_. The elimination rate constant (λ_z_) was obtained from the terminal slope of the log-transformed concentration–time curve. Volume of distribution (V_d_) was calculated as dose/(AUC_0–24_*λ_z_) and the terminal half-life (T_1/2_) as ln(2)/λ_z_. Secondly, PK parameters of isavuconazole were estimated via maximum a posteriori prediction using the population PK model developed by Desai et al. (NONMEM version 7.5; ICON Development Solutions, Gaithersburg, Maryland, USA) [28]. The population PK model by Desai et al. was developed based on isavuconazole data from nine phase I trials and the phase III SECURE trial. It is a two-compartment model with interindividual variability in CL, the volume of distribution in the peripheral compartment (V_p_) and intercompartmental clearance (Q), describing the time course of total plasma isavuconazole concentrations. The model includes covariate effects of race (i.e., “Asian” or “predominantly Caucasian”) on CL, and the effect of body mass index and the difference between healthy individuals and patients with invasive fungal infections on V_p_. The AUC_0–24_ and AUC_0–24_ at steady state (AUC_0–24,ss_) for a maintenance dose of 300 mg q24h (patient 1) or 200 mg q24h (patient 2) were derived from the cumulative AUCs, which were calculated using NONMEM by integrating the amounts of the drug in a dummy compartment. The isavuconazole distribution (T_1/2,α_) and elimination half-lives (T_1/2,β_) were calculated from the individual PK parameter estimates. The PK parameters of the two patients with ECMO support were compared with those of a typical Caucasian patient in the population PK model by Desai et al. (i.e., race: predominantly Caucasian; health status: patient with an invasive fungal infection; body mass index: 24.8 kg/m^2^) [28]. Thirdly, PK parameters were calculated by means of maximum a posteriori prediction using the population PK model developed by Perez et al. [17]. This population PK model was developed based on data from 18 critically ill patients receiving isavuconazole for the treatment of CAPA. It is a one-compartment model with interindividual variability in CL and V_d_. The model includes a covariate effect of RRT on CL. The PK parameters of the two patients with ECMO support were calculated as described for the second method and were compared with those of a typical patient without RRT in the population PK model by Perez et al. [17].

### 2.4. Narrative Literature Review

To review the available literature on isavuconazole exposure in adult patients with ECMO support, a PubMed search for English and Dutch manuscripts, published up to March 2023, was conducted by combining the terms “Isavuconazole” and “ECMO”. Case reports/series and observational PK studies reporting on the exposure to isavuconazole during ECMO support were included.

### 2.5. Ethics

Informed consent was obtained from the patients’ closest relative in accordance with the local ethics committee policy.

## 3. Results

### 3.1. Case Descriptions

#### 3.1.1. Case 1

A 61-year-old man (81 kg) with a history of diabetic nephropathy for which a deceased donor renal transplantation had been performed, was admitted to our hospital because of COVID-19-associated respiratory failure. On day 4, the patient was transferred to the ICU due to progressive respiratory failure requiring mechanical ventilation. Piperacillin–tazobactam therapy (4 g q6h) was initiated for the treatment of a pneumonia caused by *Serratia marcescens* (day 6) and switched to meropenem due to a lack of clinical response and identification of *Pseudomonas aeruginosa* via bronchoalveolar lavage (BAL) culture (day 11). On day 7 of hospital admission, CAPA was suspected based on clinical (pulmonary infiltrate) and mycological (serum galactomannan index of 0.7 and culture of *A. fumigatus* spp. *complex* in BAL) evidence. Isavuconazole therapy was initiated intravenously with a loading dose of 200 mg q8h during the first 48 h, followed by a maintenance dose of 200 mg q24h. The plasma isavuconazole C_min_ (±24 h post-dose), determined on the fourth treatment day, was 1.4 mg/L. This C_min_ exceeded the EUCAST breakpoint of 1 mg/L for *A. fumigatus*, *A. flavus* and *A. terreus.* Yet, C_min_ exposure above 2 mg/L, as targeted in our center, was not achieved. Therefore, the maintenance dose was increased to 300 mg q24h on the sixth day of treatment [10,12,13]. On the same day, veno-venous ECMO support (Hilite 7000LT oxygenator, Deltastream DP3 blood pump, Medos Medizintechnik, Stolberg, Germany; polyvinylchloride (PVC) tubing with Rheoparin coating) was started as the patient’s respiratory function deteriorated despite maximal ventilatory settings. On the fourth day of concomitant isavuconazole and ECMO treatment, the isavuconazole C_min_ was 2.2 mg/L. Isavuconazole therapy was switched to liposomal amphotericin B (3 mg/kg q24h) two days later due to resistance to isavuconazole (i.e., minimally inhibitory concentration of 4.0 mg/L). Antifungal therapy and ECMO support were continued until the patient died, 33 days after hospital admission.

#### 3.1.2. Case 2

A 52-year-old woman (67 kg) was hospitalized because of anorexia, nausea, diarrhea and respiratory failure secondary to a COVID-19 infection. She had a history of a living donor renal transplantation due to chronic glomerulonephritis. Despite maximal oxygen therapy, the patient’s respiratory function deteriorated, and she was admitted to the ICU for non-invasive respiratory support (Optiflow™ Nasal High Flow therapy) and— subsequently—mechanical ventilation. Due to progressive respiratory failure despite maximal ventilatory support, veno-venous ECMO support (Hilite 7000LT oxygenator, Medos Deltastream DP3 blood pump, Medos Medizintechnik, Stolberg, Germany; PVC tubing with Rheoparin coating) was initiated on day 16 of hospital admission. Furthermore, continuous veno-venous hemofiltration (CVVH) was started seven days after ECMO initiation. Total ECMO circuit exchanges were performed on days 12 and 27, and exchange of solely the membrane oxygenator (A.L.ONE, Eurosets, Medolla, Italy) was performed on day 17 of ECMO support. One day after the last total circuit exchange (i.e., day 28 of ECMO support; day 43 of admission), intravenous antifungal therapy with isavuconazole was initiated for the treatment of probable CAPA (tracheobronchitis and serum galactomannan index of 5.9 in BAL) according to the standard dosing regimen. After nine days of isavuconazole therapy, the maintenance dose of 200 mg q24h was increased to 400 mg q24h based on a C_min_ of 0.8 mg/L. Following the dosage increase, a C_min_ of 1.0 mg/L was measured on day 14 of antifungal treatment. Besides antifungal treatment, multiple empirical and targeted antibacterial therapy courses were administered during the patient’s admission, including moxifloxacin for suspected bacterial pneumonia and meropenem/levofloxacin for *Moraxella osloensis* bacteremia. Unfortunately, the patient’s clinical status did not improve despite maximal supportive therapy with ECMO, CVVH and antimicrobial therapy, and she died after 58 days of hospitalization.

### 3.2. Pharmacokinetics

Extensive PK sampling was performed on day 7 of isavuconazole treatment (i.e., day 2 of concomitant ECMO and isavuconazole treatment) in patient 1 and on day 4 of isavuconazole treatment (i.e., day 4 of concomitant treatment) in patient 2. On the PK sampling day, patient 2 received a standard 200 mg daily maintenance dose of isavuconazole, whereas in patient 1, the maintenance dose was increased to 300 mg q24h one day prior to sample collection.

Patient characteristics and details of isavuconazole therapy and ECMO support are summarized in Table 1.

Total isavuconazole trough and peak (i.e., ±30 min after the end of infusion) concentrations were 1.5 mg/L and 2.9 mg/L in patient 1 and 1.0 mg/L and 2.8 mg/L in patient 2, respectively. The PK parameters, estimated using non-compartmental analysis and the population PK models of Desai et al. [28] and Perez et al. [17], are summarized in Table 2. AUC_0–24_, calculated using the three different methods, ranged from 46.6 to 52.7 mg × h/L and from 29.8 to 33.4 mg × h/L for patients 1 and 2, respectively. CL values, estimated using the population PK model of Desai et al., were higher for both patients compared with the CL of a typical Desai patient. When using the model by Perez et al., the CL of patient 2 was higher than that of a typical Perez patient, whereas the CL of patient 1 was approximately the same as the CL of a typical patient.

**Figure 1:**
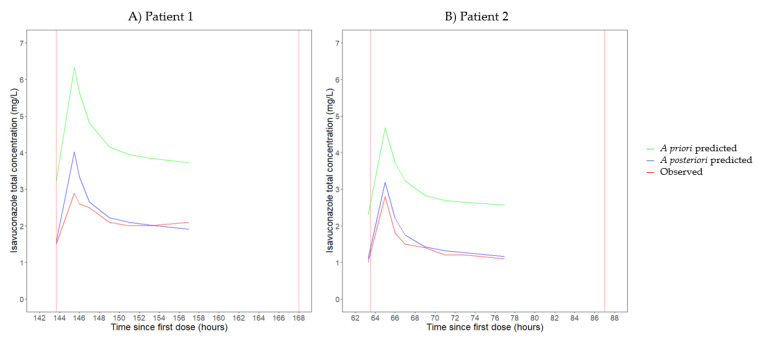
Concentration–time curves for observed, a priori predicted and a posteriori predicted total isavuconazole concentrations of patient 1 (**A**) and patient 2 (**B**). A priori and a posteriori predictions were performed using the population pharmacokinetic model of Desai et al. [28].

Figure 1A,B represent the concentration–time profiles for the observed, a priori predicted (i.e., based on the covariates in the population PK model by Desai et al.) and a posteriori predicted (i.e., based on the covariates and the observed concentrations) total isavuconazole concentrations of patients 1 and 2, respectively. Overall, an adequate fit between the observed and a posteriori predicted concentrations was documented, except that the observed peak concentration in patient 1 was lower compared with the a posteriori predicted peak concentration. When only the model covariates were taken into account (i.e., a priori predictions), the predicted concentrations were consistently higher than the observed concentrations for both patients.

**Figure 2:**
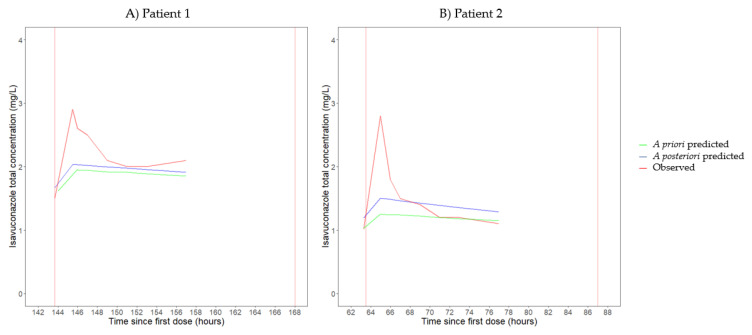
Concentration–time curves for observed, a priori predicted and a posteriori predicted total isavuconazole concentrations of patient 1 (**A**) and patient 2 (**B**). A priori and a posteriori predictions were performed using the population pharmacokinetic model of Perez et al. [17].

In Figure 2A,B, the a priori and a posteriori predicted concentrations based on the population PK model of Perez et al. [17] are compared with the observed isavuconazole concentrations of patients 1 and 2, respectively. For both patients, the agreement between observed and a priori or a posteriori predicted concentrations was inadequate at all timepoints, except for the trough concentrations.

### 3.3. Literature Review

The literature search yielded 14 records, of which 6 were reports on exposure to isavuconazole in critically ill adult patients supported by ECMO (Table 3). Two case reports [30,31], two retrospective studies (*n* = 5 and *n* = 4) [17,32] and two prospective observational studies (*n* = 3 and *n* = 7) [14,33] were identified. In total, isavuconazole exposure was described in 21 patients with ECMO support. Sixteen patients required ECMO support for virus-associated respiratory insufficiency (COVID-19 infection in 14 patients, influenza virus infection in 2 patients). Fourteen patients were supported by veno-venous ECMO, one patient by veno-arterial ECMO, and one patient sequentially required (i) veno-arterial ECMO, (ii) double-circuit veno-arterial and veno-pulmoarterial ECMO and (iii) veno-venous ECMO. The modality of ECMO support was not documented for five patients. The standard isavuconazole maintenance dose of 200 mg q24h was adjusted in at least five patients during ECMO support or following ECMO discontinuation based on TDM [30,31,32]. Extensive 24 h blood sampling was only performed in the study by Kriegl et al. [33], whereas the other research groups solely reported trough concentrations. C_min_ during ECMO support ranged from 0.5 mg/L to 5.0 mg/L, resulting from variable dosing regimens and sampling at variable timepoints during isavuconazole treatment. Pre- and post-membrane oxygenator concentrations were reported by Kriegl et al. and Zhao et al. [31,33].

## 4. Discussion

We performed extensive PK sampling in two adult, critically ill patients with COVID-19-associated respiratory failure requiring veno-venous ECMO support. Both patients were intravenously treated with isavuconazole for probable CAPA. Considering the lack of a rich sampling-based population PK model in critically ill patients, PK parameters of isavuconazole were calculated using three alternative PK methods. For both patients, AUC values were substantially reduced as compared to the median AUC_0–24,ss_ for—predominantly hematological—patients with invasive fungal infections included in the phase III SECURE trial (i.e., 89.6 mg × h/L) [10,28]. This finding is consistent regardless of the PK method applied. For patient 1, the AUC_0–24,ss_ was reduced even for an increased maintenance dose of 300 mg q24h. Accordingly, on the days of extensive PK sampling, the isavuconazole C_min_ for patients 1 and 2 (1.5 mg/L and 1.0 mg/L, respectively) was lower than the mean exposure documented in the SECURE trial (mean ± SD C_min_ on days 7 and 14: 2.6 ± 1.0 mg/L and 3.0 ± 1.4 mg/L, respectively) [10,13]. In both cases, the standard isavuconazole maintenance dose of 200 mg q24h was increased to target a C_min_ of 2 mg/L or higher, as advocated in our center. Following routine TDM, dosage was increased to 300 mg q24h in patient 1 based on a non-steady-state C_min_ of 1.4 mg/L (day 4 of therapy) and to 400 mg q24h on day 9 of isavuconazole therapy in patient 2 based on a concentration of 0.8 mg/L.

Drug exposure in patients supported by ECMO is the result of a complex interplay between physiologic alterations associated with critical illness, ECMO and other extracorporeal treatments [9,18]. ECMO might reduce drug exposure via two major mechanisms: (i) hemodilution resulting from circuit priming and (ii) drug sequestration into the ECMO circuit [24,25,26,27]. Hemodilution primarily affects the V_d_ of hydrophilic drugs, whereas drug sequestration is particularly relevant for lipophilic drugs with a high degree of plasma protein binding, such as isavuconazole [24,25,26,27]. In our patients, we estimated drug exposure (i.e., AUC) and PK parameters using non-compartmental analysis and two previously published population PK models. Currently, data on the PK of isavuconazole in critically ill patients are scarce [16,17], and only one population PK model has been developed in this patient population, i.e., the model by Perez et al. [17]. This model was developed based on data from critically ill patients with CAPA, including patients with ECMO, which is in accordance with the characteristics of our patients. Importantly, this model was fitted on trough concentrations only. Consequently, it is no surprise that we observed an inadequate fit between the a posteriori predicted and observed isavuconazole concentrations of both patients at all timepoints, except for the trough concentration (Figure 2A,B). Therefore, the population PK model by Perez et al. was considered inappropriate to adequately estimate the PK parameters of our patients. Furthermore, we used the population PK model by Desai et al. to estimate individual PK parameters [28]. The population PK model by Desai et al. was fitted on extensive sampling data from healthy participants in phase I clinical trials and on extensive or sparse sampling data from patients with invasive mold infections in the phase III SECURE trial [10,28,35,36,37]. Although the model by Desai et al. was not developed based on data from critically ill patients, isavuconazole concentrations in our patients were adequately predicted by this population PK model, as shown by the concentration–time profiles for observed and a posteriori predicted isavuconazole concentrations in Figure 1. For patient 1, Figure 1A displays a discrepancy between the observed and a posteriori predicted maximal concentration. This might be explained by an increased interindividual variability in V_d_ in critically ill patients compared with the healthy or less severely ill participants included in the study by Desai et al. Considering the limitations of the population PK models of Perez et al. and Desai et al. concerning the PK sampling design and target population, respectively, we also calculated the PK parameters using non-compartmental analysis. Importantly, population PK models fitted on rich sampling data from critically ill patients are eagerly awaited (e.g., data from the multicenter prospective observational ICONIC study, ClinicalTrials.gov: NCT04777058).

The results of the three methods suggest that reduced AUC values in our patients were related to an increase in CL compared with the CL of a typical non-critically ill patient (i.e., 2.4 L/h) [28]. It is unlikely that this increased CL is related to alterations in renal function. For patient 1, mild to moderate renal impairment (i.e., estimated glomerular filtration rate: 46 mL/min/1.73 m^2^) was observed, which might have led to decreased—rather than increased—renal clearance [38]. Furthermore, alterations in renal function do not significantly impact total isavuconazole clearance as renal elimination of unchanged isavuconazole is minimal (i.e., <1%) [7,38,39]. For patient 2, who received CVVH during concomitant isavuconazole–ECMO therapy, it is thought that transmembrane clearance only limitedly (i.e., 0.7%) contributed to total isavuconazole clearance [40]. However, a potential impact of RRT on isavuconazole clearance cannot be ruled out as a significant increase in isavuconazole clearance was observed in critically ill patients with RRT—albeit no CVVH—in the population PK analysis by Perez et al. [17]. Although isavuconazole is predominantly metabolized by the liver, the increased estimated isavuconazole clearance in our patients is not expected to be associated with increased hepatic metabolism. Firstly, in critically ill patients, a decrease in hepatic metabolism is common owing to pathophysiological alterations such as reduced CYP450 activity or decreased hepatic blood flow resulting from systemic inflammatory response syndrome [41]. Secondly, in our patients, no CYP3A-inducing drugs were administered at the time of or within 14 days prior to PK sampling. Thirdly, we do not expect CYP3A genotypes—or the associated metabolism phenotypes—to have an impact on isavuconazole clearance. For CYP3A5, three variant alleles (i.e., *3, *6 and *7) have been identified as nonfunctional alleles [42]. In the Near Eastern population (cf., biogeographical ancestry of patients 1 and 2 in Table 1), 77% of the individuals carry two nonfunctional alleles, resulting in a poor metabolizer phenotype [43]. On the contrary, only 1.5% of the Near Eastern population is a CYP3A5 normal metabolizer (i.e., carrier of two functional CYP3A5*1 alleles) [43]. Although CYP3A5 diplotypes were not determined in our patients, they are more likely to be poor versus normal metabolizers considering the low incidence of the CYP3A5*1/*1 diplotype in the Near Eastern population [42,43]. Given the absence of a conclusive (pathophysiological) explanation for the augmented clearance in our patients, this increase might (partially) reflect an apparent increase in clearance resulting from isavuconazole sequestration into the ECMO circuit. However, further research on this hypothesis is needed (cf., Discussion, sixth paragraph).

The V_d_ estimates of the three methods should be interpreted cautiously. Firstly, direct comparison of PK estimates derived from non-compartment, one-compartment and two-compartment models is challenging. Secondly, as the model by Perez et al. was only fitted on trough concentrations, V_d_ could not be estimated correctly using this model. Thirdly, the individual volumes of distribution in the central compartment (V_c_) could not be estimated based on the model by Desai et al. as no significant interindividual variability in V_c_ had been observed in their population PK analysis [28]. Yet, as V_c_ accounts for <16% of total V_d_, we expect that alterations in V_c_—if present in our patients—would have a limited impact on total V_d_.

Isavuconazole exposure in critically ill adult patients with ECMO support has only been documented in two case reports [30,31], two retrospective studies (*n* = 5 and *n =* 4) [17,32] and two prospective observational studies (*n* = 3 and *n* = 7) [14,33]. Even the multinational prospective Antibiotic, Sedative and Analgesic Pharmacokinetics during ECMO (ASAP ECMO) study, investigating antimicrobial PK in patients receiving ECMO, did not include isavuconazole cases [44]. In a recently published prospective observational PK study, Kriegl et al. investigated the impact of ECMO on the exposure to isavuconazole in seven critically ill patients who received isavuconazole prophylaxis or therapy during ECMO support [33]. A median isavuconazole C_min_ of ≥1 mg/L and ≥2 mg/L was achieved from 24 h and 96 h, respectively, after the first administration onwards. Yet, individual concentrations below 2 mg/L were observed at all sampling times (up until the seventh treatment day). Compared with AUC_0–24_ and CL in our patients, median AUC_0–24_ (15.6 [12.2–18.9] mg × h/L) reported by Kriegl et al. was lower, and median [IQR] CL (26.9 [18.4–35.3] L/h) was markedly higher for the first 24 h of isavuconazole therapy. It should be noted that in our report, the AUC_0–24_ was calculated on day 4 (patient 2) and on day 7 (patient 1) of isavuconazole therapy. Hence, it is no surprise that the estimated AUC_0–24_ on day 1 in the study by Kriegl et al. was lower. In addition to the increased clearance, an increased V_d_ (median [IQR]: 6.1 [3.9–8.3] L/kg) was documented by Kriegl et al., which was ascribed to ECMO support, besides systemic inflammation and higher body weight. However, no sequestration of isavuconazole into the ECMO circuit was observed as pre- and post-membrane oxygenator concentrations were identical to arterial isavuconazole concentrations. Noteworthy, the potential impact of saturation of the ECMO circuit binding sites by (lipophilic) drugs cannot be excluded as the age of the ECMO circuit at the time of membrane oxygenator sampling was not specified [26,27]. Perez et al. included in their population PK analysis a subset of five patients with ECMO support [17]. In their total critically ill population, they observed an increased mean clearance, especially in patients with RRT. RRT was retained as a statistically significant covariate on CL in the final population PK model, whereas ECMO had no significant impact on isavuconazole CL or V_d_. Importantly, their analysis was based solely on trough concentrations. Moreover, details on ECMO support and isavuconazole therapy were not documented for the subgroup of patients with ECMO support. In the case reports by Miller et al. and Zhao et al., an isavuconazole C_min_ below 2 mg/L was observed during ECMO support when the standard maintenance dose was administered, in accordance with isavuconazole exposure in our two cases [30,31]. Dosage was increased to 400 mg q24h [30] or 200 mg q12h [31], respectively, which subsequently resulted in a C_min_ above 2 mg/L. Zurl et al. included a subset of three patients with concomitant ECMO and RRT in their prospective observational study of 32 critically ill and non-critically ill patients treated with isavuconazole [14]. In all three ECMO patients, the isavuconazole C_min_ was below 2 mg/L during concomitant ECMO-RRT support when isavuconazole was administered according to the standard dosage regimen. In patient 2, the concentration increased to 2.2 mg/L after ECMO discontinuation (RRT only), whereas in patient 3, C_min_ remained below 0.9 mg/L after ECMO discontinuation (RRT only). In the case series by Mertens et al. (*n* = 4) highly variable isavuconazole C_min_ values were reported during ECMO support (range: 0.6–4.3 mg/L), which resulted from both standard and increased isavuconazole dosing regimens [32].

The available reports on isavuconazole in patients supported by ECMO, including our cases, are characterized by a high degree of heterogeneity in terms of patient characteristics, age of the ECMO circuit, duration of isavuconazole therapy and isavuconazole dosage regimen at the time of PK sampling. Furthermore, due to the absence of a (matched) control group including critically ill patients without ECMO support, the incremental effect of ECMO versus critical illness on the PK of isavuconazole cannot be deduced from the published reports. A population PK analysis, pooling PK data from critically ill patients with and without ECMO support, would enable assessment of the impact of covariates such as ECMO on the (variability in) the PK of isavuconazole. Pending robust data, TDM of isavuconazole is warranted in critically ill patients, including those supported by ECMO.

In addition to the aforementioned limitations concerning our case report design and the lack of an appropriate population PK model for critically ill patients, the following three points of concern might further challenge the interpretation of the results of the current and previous isavuconazole PK studies. Firstly, isavuconazole exposure targets for efficacy and safety have not been robustly established yet as data from the SECURE phase III clinical trial did not reveal concentration-dependent relationships for efficacy and toxicity [10,13,28]. However, a C_min_ of 2 mg/L and higher is often targeted in clinical practice based on the mean exposure attained in the SECURE trial, in which efficacy against invasive mold infections was demonstrated [10,12,13]. Moreover, a C_min_ of 1 mg/L is often advocated as the minimum threshold for efficacy based on the EUCAST susceptibility breakpoints for *A. fumigatus*, *A. flavus* and *A. terreus* [19]. Secondly, unbound—i.e., physiologically active—isavuconazole concentrations have not been reported to date. Nevertheless, critically ill patients often display a high variability in serum albumin levels (e.g., in the present study, 25.7 g/L and 44.8 g/L in patients 1 and 2, respectively). This interindividual and interoccasion variability in serum albumin concentrations might influence the PK of highly protein-bound drugs such as isavuconazole [9,18]. Decreases in circulating albumin might cause a temporary increase in the unbound isavuconazole fraction, subsequently resulting in a higher distribution and clearance. Of note, no clinical exposure targets for the unbound isavuconazole fraction have been established yet. Thirdly, the long elimination half-life of isavuconazole (i.e., 110–115 h) further challenges the interpretation of TDM results for this triazole [7]. Plasma concentrations measured at non-steady-state conditions might lead to inadequate dose adjustments. This limitation might be mitigated by performing TDM for isavuconazole supported by model-informed precision dosing as this TDM approach enables non-steady-state dosing calculations.

## 5. Conclusions

We reported PK parameters and exposure metrics of isavuconazole in two critically ill adult patients supported by ECMO. The AUC values were markedly decreased in these two patients in comparison with AUC values previously reported in non-critically ill patients. The reduced AUC in both patients was related to an increased CL of isavuconazole. The altered isavuconazole exposure in our patients emphasizes the increased risk for PK changes and the need for dose optimization in critically ill patients [9,16,18,33]. However, it remains debatable whether the reduced exposure observed during ECMO support in our case series is attributable to ECMO itself or to critical illness. Additional research, including rich sampling-based population PK modeling for isavuconazole in critically ill ECMO and non-ECMO patients, is needed to elucidate the incremental effect of ECMO versus critical illness on the PK of isavuconazole. Pending robust outcomes of these studies, TDM is recommended in critically ill patients, regardless of ECMO support, to ensure efficacy.

## Figures and Tables

**Table 1:** Patient characteristics, treatment data and isavuconazole exposure metrics in two patients concomitantly treated with isavuconazole and extracorporeal membrane oxygenation.

	Patient 1	Patient 2
**Demographics**		
Age, years	61	52
Sex	Male	Female
Biogeographical ancestry ^a^	Near Eastern	Near Eastern
Body weight, kg	81.3	67.0
BMI, kg/m^2^	24.3	27.5
**Clinical characteristics**		
Length of ICU stay ^b^, days	30	48
Host factors ^c^	COVID-19 infection needing intensive care	COVID-19 infection needing intensive care
APACHE II score on admission	18	28
SOFA score ^d^	10	15
Serum albumin ^d^, g/L	25.7	44.8
ALT ^d^, U/L	15	167
AST ^d^, U/L	14	152
eGFR (CKD-EPI) ^d^, mL/min/1.73 m^2^	46	NA ^e^
CRRT during isavuconazole therapy	No	Yes
Total duration of CRRT, days	NA	36
Duration of CRRT until extensive PK sampling, days	NA	24
Deceased during ICU admission	Yes	Yes
**ECMO support**		
Indication of ECMO support	COVID-19-associated ARDS	COVID-19-associated ARDS
ECMO modality	Veno-venous	Veno-venous
ECMO oxygenator	Medos Hilite 7000LT	Medos Hilite 7000LT/Eurosets A.L.ONE
ECMO blood pump	Medos Deltastream DP3	Medos Deltastream DP3
Priming solution	Plasma-Lyte A	Plasma-Lyte A
Priming volume, mL	670	670
Total duration of ECMO support, days	22	43
Duration of ECMO support at initiation of isavuconazole, days	NA	28
Duration of ECMO support until extensive PK sampling, days	1	31
ECMO circuit exchanges ^f^	No exchanges	Days 12, 17 *, 27
**Isavuconazole therapy**		
Indication	Probable CAPA ^g^	Probable CAPA ^g^
Therapy duration, days	10	16
Duration of isavuconazole therapy until extensive PK sampling, days	7	4
Maintenance dose on the extensive PK sampling day	300 mg q24h ^h^	200 mg q24h
Trough concentrations ^i^, mg/L	Day 4: 1.4 ** Day 7: 1.5 Day 9: 2.2 **	Day 4: 1.0 Day 7: 0.8 ** Day 14: 1.0 **

ALT: alanine aminotransferase; APACHE II: Acute Physiology and Chronic Health Evaluation II score; ARDS: acute respiratory distress syndrome; AST: aspartate aminotransferase; BMI: body mass index; CAPA: COVID-19-associated pulmonary aspergillosis; CKD-EPI: Chronic Kidney Disease Epidemiology Collaboration; COVID-19: coronavirus disease 2019; CRRT: continuous renal replacement therapy; ECMO: extracorporeal membrane oxygenation; eGFR: estimated glomerular filtration rate; ICU: intensive care unit; NA: not applicable; SOFA: Sequential Organ Failure Assessment. ^a^ Biogeographical ancestry was defined by the standardized biogeographic grouping system of PharmGKB [29]. The Near Eastern genetic ancestry group includes populations from Northern Africa, the Middle East and the Caucasus. For the covariate effect of race on clearance in the population pharmacokinetic model by Desai et al., both patients were classified as “predominantly Caucasian” [28]. ^b^ From admission to the intensive care unit until death. ^c^ Host factors for COVID-19-associated pulmonary aspergillosis according to the 2020 European Confederation of Medical Mycology (ECMM) and the International Society of Human and Animal Mycology (ISHAM) consensus criteria [4]. ^d^ Measured on the day of extensive pharmacokinetic sampling. ^e^ Patient 2 received continuous veno-venous hemofiltration on the day of extensive pharmacokinetic sampling. ^f^ The days of ECMO circuit exchanges were calculated in relation to the initiation of ECMO support. The asterisk (*) indicates an exchange of solely the ECMO oxygenator. ^g^ The classification was performed according to the 2020 ECMM and ISHAM consensus criteria for COVID-19-associated pulmonary aspergillosis [4]. ^h^ The standard maintenance dose of 200 mg q24h was increased to 300 mg q24h one day before extensive pharmacokinetic sampling. ^i^ The days of trough concentration determination were calculated in relation to the initiation of isavuconazole therapy. The double asterisk (**) indicates trough concentrations that were determined via routine therapeutic drug monitoring.

**Table 2:** Pharmacokinetic parameters for patients 1 and 2, calculated using non-compartmental analysis and the population pharmacokinetic models by Desai et al. [28] and Perez et al. [17].

	Sampling and Isavuconazole Dosing		PK Parameters ^a^					
	Number of Samples (n)	Isavuconazole Maintenance Dose ^b^	CL (L/h)	Q (L/h)	V_d_ (L)	T_1/2_ (h)	AUC_0–24_ (mg × h/L)	AUC_0–24,ss_ (mg × h/L)
**Non-compartmental analysis**								
Patient 1	8	300 mg q24h	6.3	NA	310.8	34.2	47.8	NA
Patient 2	8	200 mg q24h	6.7	NA	212.6	21.9	29.8	NA
**Population PK model by Desai et al.** [28]								
Patient 1	8	300 mg q24h	5.4 [5.1–5.7]	34.8 [25.0–48.3]	V_c_: 49.1 V_p_: 258.3 [219.9–323.4]	T_1/2,α_: 0.74 T_1/2,β_: 44.1	52.7	55.7
Patient 2	8	200 mg q24h	7.2 [6.5–8.1]	28.3 [20.3–39.5]	V_c_: 49.1 V_p_: 342.1 [243.4–355.0]	T_1/2,α_: 0.85 T_1/2,β_: 35.6	33.4	27.7
Typical patient ^c^	NA	200 mg q24h	2.4	26.6	V_c_: 49.1 V_p_: 260.0	T_1/2,α_: 1.01 T_1/2,β_: 96.5	NA	89.3
**Population PK model by Perez et al.** [17]								
Patient 1	8	300 mg q24h	4.2 [2.9–6.2]	NA	776.5 [616.5–978.0]	128.5	46.6	62.5
Patient 2	8	200 mg q24h	7.5 [5.5–10.2]	NA	588.8 [432.7–801.3]	54.4	30.9	26.9
Typical patient ^d^	NA	200 mg q24h	4.0	NA	850.0	148.1	NA	50.2

AUC_0–24_: area under the concentration–time curve over 24 h for the pharmacokinetic sampling interval; AUC_0–24,ss_: area under the concentration–time curve over 24 h at steady state; CL: clearance; NA: not applicable; PK: pharmacokinetic; Q: intercompartmental clearance; T_1/2_: half-life; T_1/2,α_: distribution half-life; T_1/2,β_: elimination half-life; V_c_: volume of distribution in the central compartment; V_d_: volume of distribution; V_p_: volume of distribution in the peripheral compartment. ^a^ The 95% confidence intervals are indicated for the individual pharmacokinetic parameter estimates if applicable (i.e., for parameters with interindividual variability in the population pharmacokinetic models). ^b^ Isavuconazole maintenance dose at the time of extensive pharmacokinetic sampling. ^c^ Typical Caucasian patient in the population pharmacokinetic model by Desai et al. [28]. Pharmacokinetic parameters for the typical patient were derived from the parameter estimates of the final covariate model by Desai et al. for a Caucasian patient with a body mass index of 24.8 kg/m^2^. ^d^ Typical patient in the population pharmacokinetic model by Perez et al. [17]. Pharmacokinetic parameters for the typical patient were derived from the parameter estimates of the final covariate model by Perez et al. for a critically ill patient who had not received renal replacement therapy.

**Table 3:** Summary table of previously published reports (*n* = 6) on isavuconazole exposure in critically ill adult patients (*n* = 21) supported by extracorporeal membrane oxygenation.

Reference (Year)	Perez et al. (2023) [17]	Kriegl et al. (2022) [33]	Mertens et al. (2022) [32]	Miller et al. (2022) [30]	Zhao et al. (2020) [31]	Zurl et al. (2020) [14]
Study characteristics						
Study design	Single-center retrospective study	Single-center prospective observational PK study	Single-center retrospective study	Case report	Case report	Multicenter prospective observational cohort study
Number of patients	5 ^a^	7	4	1	1	3 ^b^
**Demographics**						
Age, years	Median [IQR] in total cohort: 65 [56–70]; NS for patients with ECMO	Median [IQR]: 58 [50–62]	* Patient 1: 61 * Patient 2: 59 * Patient 3: 65 * Patient 4: 38	56	26	Median [IQR] in total cohort: 60 [46–69]; NS for patients with ECMO
Sex	Total cohort: 22% female; NS for patients with ECMO	43% female	100% male	Male	Male	Total cohort: 28% female; NS for patients with ECMO
BMI, kg/m^2^	Median [IQR] in total cohort: 29.2 [25.6–31.8]; NS for patients with ECMO	Median [IQR]: 29.8 [26.9–35.2]	* Patient 1: 26.7 * Patient 2: 20.0 * Patient 3: 29.2 * Patient 4: 24.7	20.3	NS	Median [IQR] in total cohort: 24.6 [23.3–28.5]; NS for patients with ECMO
**Clinical characteristics**						
Host factors ^c^	COVID-19 infection needing intensive care (*n* = 5)	COVID-19 infection needing intensive care (*n* = 6); NS (*n* = 1)	**Patients 1, 3, 4** COVID-19 infection needing intensive care **Patient 2** Solid organ Tx	Immunosuppressive therapy	None	**Patients 1, 2** Influenza virus infection needing intensive care **Patient 3** NS
CRRT during ICU admission	NS for patients with ECMO	None of the included patients received CRRT	**Patients 1, 3** Concomitant ISA-CRRT-ECMO therapy **Patients 2, 4** No CRRT	No CRRT	Concomitant ISA-CRRT-ECMO therapy	**Patient 1** Concomitant ISA-CRRT-ECMO therapy **Patient 2** Concomitant ISA-CRRT-ECMO therapy (6 days) → ISA-CRRT therapy **Patient 3** Concomitant ISA-ECMO therapy (11 days) → ISA-CRRT-ECMO therapy (3 days) → ISA-CRRT therapy
**ECMO support**						
Indication of ECMO support	COVID-19-associated ARDS (*n* = 5)	COVID-19-associated ARDS (*n* = 6); cardiac arrest during cardiac surgery (*n* = 1)	**Patients 1, 3, 4** COVID-19-associated ARDS **Patient 2** Respiratory insufficiency due to complications after lung Tx	Respiratory insufficiency in patient with aspergilloma	ARDS and cardiogenic shock secondary to pulmonary blastomycosis (veno-arterial)/acute primary pulmonary allograft dysfunction (veno-venous)	**Patients 1, 2** Influenza-associated ARDS **Patient 3** ARDS after major cardiac surgery
ECMO modality	NS	Veno-venous (*n* = 6); veno-arterial (*n* = 1)	Veno-venous (*n* = 4)	Veno-venous	Veno-arterial → veno-arterial and veno-pulmoarterial → veno-venous	Veno-venous (*n* = 3)
Composition of ECMO circuit	NS	Novalung XLUNG kit 230 membrane oxygenator (Xenios AG); Deltastream DP3 blood pump (Medos Medizintechnik AG)	NS	Quadrox-iD adult membrane oxygenator (Maquet); Jostra Rotaflow pump (Maquet); PVC tubing with phosphorylcholine coating (LivaNova)	Cardiohelp v7 polymethylpentene membrane oxygenator and centrifugal pump (Maquet); PVC tubing	**Patients 1, 2** Novalung iLA activve system (Xenios AG) **Patient 3** NS
Circuit exchanges ^d^	NS	NS	**Patients 1, 4** No exchanges **Patient 2** Day 8 * Patient 3 Days 31, 49, 57, 64 *	Day 23	* Day 33: addition of veno-pulmoarterial ECMO circuit * Day 152: single veno-venous ECMO circuit	NS
Duration of ECMO at initiation of ISA therapy, days	NS	NS	* Patient 1: 16 * Patient 2: 6 * Patient 3: 24 * Patient 4: NA	13	64	NS
**Antifungal therapy**						
Indication of antifungal therapy	CAPA (*n* = 5)	Antifungal prophylaxis in patients with COVID-19 infection (*n* = 6); probable IPA (*n* = 1)	**Patients 1, 4** Probable CAPA **Patient 2** Probable IPA **Patient 3** Proven CAPA	Aspergilloma (*A. fumigatus*)	Pulmonary blastomycosis (*Blastomyces dermatitidis)*	**Patients 1,3** IPA **Patient 2** Probable intra-abdominal *Candida parapsilosis* infection
Choice of antifungal therapy	ISA IV/PO No information on previous/subsequent antifungal therapies in patients with ECMO.	ISA IV No information on previous/subsequent antifungal therapies.	**Patient 1** VRC IV (8 days) → VRC IV + L-AmB IV + ABLC nebulization (10 days) → ISA IV + L-AmB IV + ABLC nebulization (9 days) **Patient 2** ISA IV **Patient 3** L-AmB IV (21 days) → Caspofungin IV + ISA IV (10 days) → L-AmB IV (36 days) → L-AmB IV + VRC IV (9 days) → VRC IV (82 days) → L-AmB IV (4 days) **Patient 4** VRC IV (7 days) → ISA IV (14 + 25 days)	VRC IV (20 days) → ISA IV	L-AmB IV (5 mg/kg q24h → 7.5 mg/kg q24h → 10 mg/kg q24h) + L-AmB nebulization (25 mg q12h) + ISA IV (from day 67 of antifungal therapy onwards)	**Patient 1** VRC IV → ISA IV **Patient 2** Caspofungin IV → ISA IV **Patient 3** NS
ISA dosing regimen	Total cohort: LD: 200 mg q8h (72h) in 6 patients/ MD (mean ± SD): 264 ± 79 mg q24h; NS for patients with ECMO	LD: 200 mg q8h (48h)/MD: 200 mg q24h	**Patient 1** LD: 200 mg q8h (48h)/MD: 200 mg q12h (3 days) → 200–150 mg (1 day) → 150 mg q12h (3 days) **Patient 2** LD: 200 mg q8h (48h)/MD: 200 mg q24h **Patient 3** LD: 200 mg q8h (48h)/MD: 200 mg q24h (2 days) → 200 mg q8h (6 days) **Patient 4 (course 1) ^e^** LD: 200 mg q8h (48h)/MD: 200 mg q24h (1 day) → 200 mg q18h (3 days) → 200 mg q12h (1 day) → 200 mg q24h (3 days) → 200 mg q12h (3 days) **Patient 4 (course 2)** LD: 200 mg q8h (48h)/MD: 200 mg q24h (6 days) → 400 mg q24h (17 days)	LD: 200 mg q8h (48h)/MD: 200 mg q24h (18 days) → 400 mg q24h (14 days)	LD: NS/MD: 200 mg q24h (9 days) → 200 mg q12h (115 days)	LD: 200 mg q8h (48h)/MD: 200 mg q24h
Duration of ISA therapy, days	NS	Median [IQR]: 11 [5–18]	* Patient 1: 9 * Patient 2: 7 * Patient 3: 10 * Patient 4: 14/25	34	124	**Patient 1** 14 **Patient 2** NS **Patient 3** 18
**Pharmacokinetics**						
PK sampling	Median [IQR] time to first C_min_ measurement in total cohort: 5 [4.3–7.5] days; NS for patients with ECMO	* Blood samples from arterial line at 2h, 4h, 8h, 12h, 18h, 24h, 48h, 72h, 96h, 120h, 144h, 168h after first ISA dose (*n* = 64) * Samples from in- and outflow line of membrane oxygenator (*n* = 27)	**Patient 1** C_min_ on days 5 and 6 after ISA initiation (ECMO + CRRT) **Patient 2** C_min_ on day 7 (ECMO) **Patient 3** C_min_ on days 2, 4, 8 and 10 (ECMO + CRRT) **Patient 4 (course 1)** C_min_ on day 7 (ECMO) **Patient 4 (course 2)** C_min_ on day 6 after ISA re-initiation (no ECMO)	C_min_ on days 5, 17 after ISA initiation and days 5, 8 after dosage adjustment	* C_min_ on days 8, 23 and 105 after ISA initiation * Pre- and post-oxygenator concentrations on day 23	**Patient 1** C_min_ on day 12 after ISA initiation (ECMO + CRRT) Patient 2 * C_min_ on days 1 and 4 after ISA initiation (ECMO + CRRT) * C_min_ after ECMO discontinuation (day of ISA therapy NS) (CRRT) **Patient 3** Daily C_min_ measurements during ISA-ECMO, ISA-ECMO-CRRT and ISA-CRRT therapy
Duration of ISA therapy/ECMO support at sampling day(s), days	* Median [IQR] time to first C_min_ measurement in total cohort: 5 [4.3–7.5] days; NS for patients with ECMO * Duration of ECMO support at sampling day(s): NS	1–7 days/NS	**Patient 1** 5, 6 days/20, 21 days **Patient 2** 7 days/13 days **Patient 3** 2, 4, 8, 10 days/27, 29, 33, 35 days **Patient 4 (course 1)** 7 days/2 days **Patient 4 (course 2)** 6 days/NA	5, 17, 25, 28 days/18, 30, 38, 41 days	8, 23, 105 days/71, 86, 168 days	**Patient 1** 12 days/NS Patient 2 * 1, 4 day(s)/NS * NS/NA **Patient 3** Daily measurements
Reported ISA concentrations	* Median [IQR] C_min_ in total cohort: 1.87 [1.29–2.25] mg/L; NS for patients with ECMO	* Median [IQR] C_min,24h_/C_min,48h_/C_min,72h_/C_min,168h_: 1.09 [0.92–1.30]/0.99 [0.94–1.51]/1.21 [1.03–2.07]/2.81 [2.09–3.76] mg/L * Significant correlation between pre- and post-membrane oxygenator ISA concentrations and between post-membrane oxygenator and arterial concentrations	Patient 1 * C_min,d5_: 4.3 mg/L * C_min,d6_: 5.0 mg/L **Patient 2** C_min,d7_: 3.1 mg/L Patient 3 * C_min,d2_: 0.7 mg/L * C_min,d4_: 0.6 mg/L * C_min,d8_: 1.1 mg/L * C_min,d10_: 1.7 mg/L **Patient 4 (course 1)** C_min,d7_: 0.8 mg/L **Patient 4 (course 2)** C_min,d6_: 0.7 mg/L	* C_min,d5_: 1.7 mg/L * C_min,d17_: 0.7 mg/L * C_min,d25_: 3.7 mg/L * C_min,d28_: 2.9 mg/L	**ISA 200 mg q24h— Double-circuit veno-arterial and veno-pulmoarterial ECMO** C_min,d8_: 1.9 mg/L **ISA 200 mg q12h— Double-circuit veno-arterial and veno-pulmoarterial ECMO** C_min_/pre-/post-oxygenator concentration day 23: 4.1/3.7/3.4 mg/L **ISA 200 mg q12h – Veno-venous ECMO** C_min,d105_: 4.7 mg/L	**Patient 1** C_min,d12_: 1.79 mg/L (ECMO + CRRT) Patient 2 * C_min,d1_: 0.74 mg/L (ECMO + CRRT) * C_min,d4_: 0.57 mg/L (ECMO + CRRT) * C_min_ after ECMO discontinuation: 2.44 mg/L (CRRT) Patient 3 * Median C_min_: 1.7 mg/L (ECMO) * Median C_min_: 0.8 mg/L (ECMO + CRRT) * Median C_min_: <0.9 mg/L (CRRT)
Results of PK analysis	* No significant effect of ECMO on V_d_ or CL in a population PK analysis (nonlinear mixed-effects modeling)	* Median ^f^ [IQR] CL: 26.9 [18.4–35.3] L/h * Median ^f^ [IQR] V_d_: 6.1 [3.9–8.3] L/kg * Median ^f^ [IQR] AUC_0–24_: 15.6 [12.2–18.9] mg × h/L	NA	NA	NA	NA

*A.*: *Aspergillus*; ABLC: amphotericin B lipid complex; ARDS: acute respiratory distress syndrome; AUC_0–24_: area under the concentration–time curve over 24 h; BMI: body mass index; CAPA: COVID-19-associated pulmonary aspergillosis; CL: clearance; C_min_; trough concentration; COVID-19: coronavirus disease 2019; CRRT: continuous renal replacement therapy; ECMO: extracorporeal membrane oxygenation; ICU: intensive care unit; IPA: invasive pulmonary aspergillosis; IQR: interquartile range; ISA: isavuconazole; IV: intravenous(ly); L-AmB: liposomal amphotericin B; LD: loading dose; MD: maintenance dose; NA: not applicable; NS: not specified; PK: pharmacokinetic(s); PO: per os; PVC: polyvinylchloride; SD: standard deviation; Tx: transplantation; V_d_: volume of distribution; VRC: voriconazole. ^a^ In the retrospective study, 18 adult patients treated with isavuconazole were included, of whom 5 were supported by extracorporeal membrane oxygenation. ^b^ In the observational cohort study, 32 adult patients treated with isavuconazole were included, of whom 3 were supported by extracorporeal membrane oxygenation. ^c^ Host factors included a recent history of neutropenia, hematologic malignancy, allogeneic stem cell transplantation, solid organ transplantation, immunosuppressive therapy (i.e., T- and B-cell immunosuppressants and prolonged use of corticosteroids) and inherited severe immunodeficiency according to the documented host factors for probable invasive fungal diseases by the European Organization for Research and Treatment of Cancer and the Mycoses Study Group Education and Research Consortium [34], a COVID-19 infection needing intensive care according to the 2020 European Confederation of Medical Mycology and the International Society of Human and Animal Mycology consensus criteria for COVID-19-associated pulmonary aspergillosis [4] and an influenza virus infection needing intensive care according to an expert-based case definition for influenza-associated pulmonary aspergillosis [3]. ^d^ The days of ECMO circuit exchanges were calculated in relation to the initiation of ECMO support. The asterisk (*) indicates an exchange of solely the membrane oxygenator. ^e^ The isavuconazole therapy was interrupted for four days. ^f^ Calculated by non-compartmental pharmacokinetic analysis for the first 24 h of isavuconazole therapy. The reported volume of distribution was expressed as L/kg instead of L (confirmed by Prof. Dr. R. Krause in written communication).

## Data Availability

All data relevant to the case report are included in the manuscript. Additional data are available from the authors upon reasonable request.
